# Neurosteroid Levels in GBA Mutated and Non-Mutated Parkinson’s Disease: A Possible Factor Influencing Clinical Phenotype?

**DOI:** 10.3390/biom14081022

**Published:** 2024-08-17

**Authors:** Francesco Cavallieri, Chiara Lucchi, Sara Grisanti, Edoardo Monfrini, Valentina Fioravanti, Giulia Toschi, Giulia Di Rauso, Jessica Rossi, Alessio Di Fonzo, Giuseppe Biagini, Franco Valzania

**Affiliations:** 1 Neurology Unit, Neuromotor & Rehabilitation Department, Azienda USL-IRCCS di Reggio Emilia, 42123 Reggio Emilia, Italy; francesco.cavallieri@ausl.re.it (F.C.); valentina.fioravanti@ausl.re.it (V.F.); giulia.toschi@ausl.re.it (G.T.); giulia.dirauso@ausl.re.it (G.D.R.); jessica.rossi@ausl.re.it (J.R.); franco.valzania@ausl.re.it (F.V.); 2 Department of Biomedical, Metabolic and Neural Sciences, University of Modena and Reggio Emilia, 41125 Modena, Italy; lucchi.chiara86@gmail.com; 3 Clinical and Experimental Medicine PhD Program, University of Modena and Reggio Emilia, 41121 Modena, Italy; grisanti.sara@gmail.com; 4 Neurology Unit, Fondazione IRCCS Ca’ Grande Ospedale Maggiore Policlinico, 20122 Milan, Italy; edoardo.monfrini@unimi.it (E.M.); alessio.difonzo@policlinico.mi.it (A.D.F.)

**Keywords:** GBA, glucocerebrosidase, neurosteroids, Parkinson’s disease, psychiatric disorders

## Abstract

Neurosteroids are pleiotropic molecules involved in various neurodegenerative diseases with neuroinflammation. We assessed neurosteroids’ serum levels in a cohort of Parkinson’s Disease (PD) patients with heterozygous glucocerebrosidase (GBA) mutations (GBA-PD) compared with matched cohorts of consecutive non-mutated PD (NM-PD) patients and healthy subjects with (GBA-HC) and without (NM-HC) GBA mutations. A consecutive cohort of GBA-PD was paired for age, sex, disease duration, Hoehn and Yahr stage, and comorbidities with a cohort of consecutive NM-PD. Two cohorts of GBA-HC and HC were also considered. Clinical assessment included the Movement Disorder Society revision of the Unified Parkinson’s Disease Rating Scale (MDS-UPDRS) and the Montreal Cognitive Assessment (MoCA). Serum samples were processed and analyzed by liquid chromatography coupled with the triple quadrupole mass spectrometry. Twenty-two GBA-PD (males: 11, age: 63.68), 22 NM-PD (males: 11, age: 63.05), 14 GBA-HC (males: 8; age: 49.36), and 15 HC (males: 4; age: 60.60) were studied. Compared to NM-PD, GBA-PD showed more hallucinations and psychosis (*p* < 0.05, Fisher’s exact test) and higher MDS-UPDRS part-II (*p* < 0.05). Most of the serum neurosteroids were reduced in both GBA-PD and NM-PD compared to the respective control cohorts, except for 5α-dihydroprogesterone. Allopregnanolone was the only neurosteroid significantly lower (*p* < 0.01, Dunn’s test) in NM-PD compared to GBA-PD patients. Only in GBA-PD, allopregnanolone, and pregnanolone levels correlated (Spearman) with a more severe MDS-UPDRS part-III. Allopregnanolone levels also negatively correlated with MoCA scores, and pregnanolone levels correlated with more pronounced bradykinesia. This pilot study provides the first observation of changes in neurosteroid peripheral levels in GBA-PD. The involvement of the observed changes in the development of neuropsychological and motor symptoms of GBA-PD deserves further attention.

## 1. Introduction

Parkinson’s disease (PD) is a multifaceted, heterogeneous neurological disorder for which the deficit in dopaminergic neurotransmission represents the core but not the unique pathophysiological mechanism involved [1,2,3]. Glucocerebrosidase (GBA) gene mutations strongly contribute to the development and progression of the disease [4,5]. Compared to non-mutated PD (NM-PD), the GBA-PD phenotype is characterized by earlier onset, a faster disease progression, and a prominent non-motor burden, particularly for psychiatric disorders (especially psychosis) [5,6,7]. The mechanisms responsible for this PD phenotype are still yet to be disclosed.

Some evidence suggests a possible link between neurosteroids (NSs) and PD [8,9,10,11]. An in vivo positron emission tomography (PET) imaging study detected an increased binding of (R)-[11C]PK11195, a prototypical translocator protein 18 kDa (TSPO) radiotracer, to the TSPO in GBA carriers without PD compared to age-matched healthy controls (HC), suggesting that this change could be an early event in GBA-PD [12]. TSPO mediates the first step leading to the synthesis of NSs [13,14]. These molecules are produced by neurons and glial cells and play an important role in modulating the central nervous system (CNS) functions by regulating neuronal growth, brain development, synapse formation, neural transmission, myelination, neurogenesis, dendritic growth, neuronal survival, and neuroinflammation [8,13,14]. Interestingly, dysregulation in NSs is emerging as a factor contributing to psychiatric disorders, including anxiety, depression, and psychosis [15,16,17].

Among NSs, allopregnanolone can potentiate the defective inhibitory response observed in prepulse inhibition (PPI) of the startle reflex, a test that evaluates the failure in information processing and response elaboration in patients with schizophrenia [18]. Interestingly, dopamine and its agonists reduce the PPI response, and this alteration was also observed in PD patients [19,20]. Psychosis is found in approximately 50% of GBA-PD, and its prevalence is higher in GBA-PD patients compared to NM-PD patients [5,6]. Whether NSs could be related to a higher risk of psychosis in PD or determine a greater predisposition to develop the disease has not yet been explored. We present here a pilot study that aimed to address these issues, evaluating both the complete serum NSs’ profile and clinical characteristics of a cohort of consecutive GBA-PD patients compared to a matched cohort of consecutive NM-PD patients and two cohorts of healthy subjects with (GBA-HC) and without (NM-HC) GBA mutations.

## 2. Results

Twenty-two GBA-PD (males: 11; females: 11; age: 63.68 years; Movement Disorder Society revision of the Unified Parkinson’s Disease Rating Scale (MDS-UPDRS) III: 36.00; Montreal Cognitive Assessment (MoCA): 22.73), 22 matched NM-PD (males: 11; females: 11; age: 63.05 years; MDS-UPDRS III: 29.68; MoCA: 21.82), 14 GBA-HC (males: 8; females: 6; age: 49.36 years; MDS-UPDRS III: 0.00; MoCA: 29.79), and 15 NM-HC (males: 4; females: 11; age: 60.60 years; MDS-UPDRS III: 0.33; MoCA: 29.40) were included in the study (Table 1).

**Figure 1:**
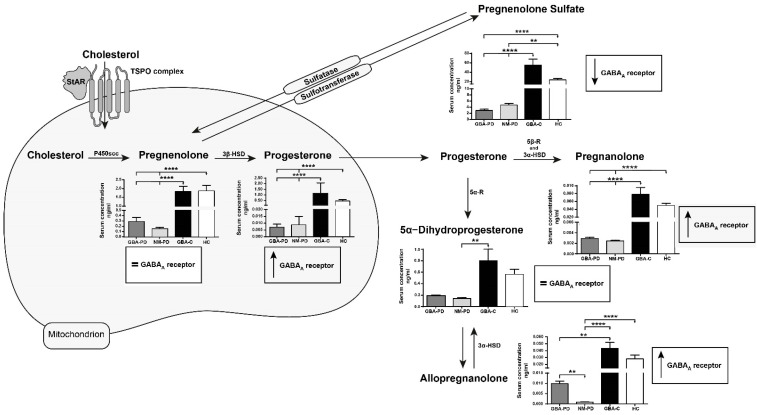
Levels of different neurosteroids (NSs) were measured in the serum of GBA-HC, NM-HC, GBA-PD, and NM-PD patients. Data obtained for the investigated NSs were ordered according to their metabolic processing. All data are represented as mean ± standard error of the mean (SEM) and were compared using the Kruskal-Wallis one-way analysis of variance (ANOVA) on ranks, followed by Dunn’s test for multiple comparisons. The inserts show that NS act differently on the γ-aminobutyric acid A (GABA_A_) receptor. Pregnenolone and 5α–DHP did not affect the activity of GABA_A_ receptors. Progesterone and its metabolites allopregnanolone and pregnanolone act in a rapid, non-genomic manner to enhance the function of GABA_A_ receptors. Pregnenolone sulfate reduces the responses of GABA_A_ receptors [13,14]. Significance: Pregnenolone, **** *p* < 0.0001 GBA-HC vs. GBA-PD and NM-PD patients, **** *p* < 0.0001 NM-HC vs. GBA-PD and NM-PD patients; Progesterone, **** *p* < 0.0001 GBA-HC vs. GBA-PD and NM-PD patients, **** *p* < 0.0001 NM-HC vs. GBA-PD and NM-PD patients; Pregnenolone sulfate, **** *p* < 0.0001 GBA-HC vs. GBA-PD and NM-PD patients, **** *p* < 0.0001 NM-HC vs. GBA-PD, ** *p* < 0.001 NM-HC vs. NM-PD; 5α–DHP, ** *p* < 0.001 GBA-HC vs. NM-PD; Pregnanolone, **** *p* < 0.0001 GBA-HC vs. GBA-PD and NM-PD patients, **** *p* < 0.0001 NM-HC vs. GBA-PD and NM-PD patients; Allopregnanolone, **** *p* < 0.0001 GBA-HC vs. NM-PD patients, ** *p* < 0.01 GBA-HC vs. GBA-PD patients, **** *p* < 0.0001 NM-HC vs. NM-PD patients, ** *p* < 0.01 GBA-PD vs. NM-PD.

Compared to GBA-PD, NM-PD, and NM-HC, GBA-HC were younger (*p* < 0.005, Tukey’s test), while no differences were found in gender distribution between the four cohorts. As shown in Figure 1, compared to both GBA-PD and NM-PD patients, GBA-HC presented significantly higher levels (*p* < 0.0001, Dunn’s test) of pregnenolone, progesterone, pregnenolone sulfate, pregnanolone, and allopregnanolone (*p* < 0.01 vs. GBA-PD, *p* < 0.0001 vs. NM-PD). At variance, 5α–dihydroprogesterone (5α–DHP) was statistically different only in NM-PD compared to GBA-HC (*p* < 0.01), who anyway were younger. The NM-HC cohort presented higher levels for almost all the evaluated NSs when compared to GBA-PD and NM-PD patients (pregnenolone, progesterone, and pregnanolone: *p* < 0.0001 vs. both cohorts of patients; pregnenolone sulfate: *p* < 0.0001 vs. GBA-PD, *p* < 0.001 vs. NM-PD), with the only exception of 5α–DHP. Notably, allopregnanolone was significantly reduced only in NM-PD (*p* < 0.001) when compared to NM-HC, and non-significantly reduced in GBA-PD vs. NM-HC. It is interesting to note that the only significant difference between GBA-PD and NM-PD cohorts was for allopregnanolone (*p* < 0.01), which was remarkably reduced in NM-PD patients (−88% vs. GBA-PD). Lastly, no differences were found between GBA-HC and NM-HC cohorts.

**Figure 2:**
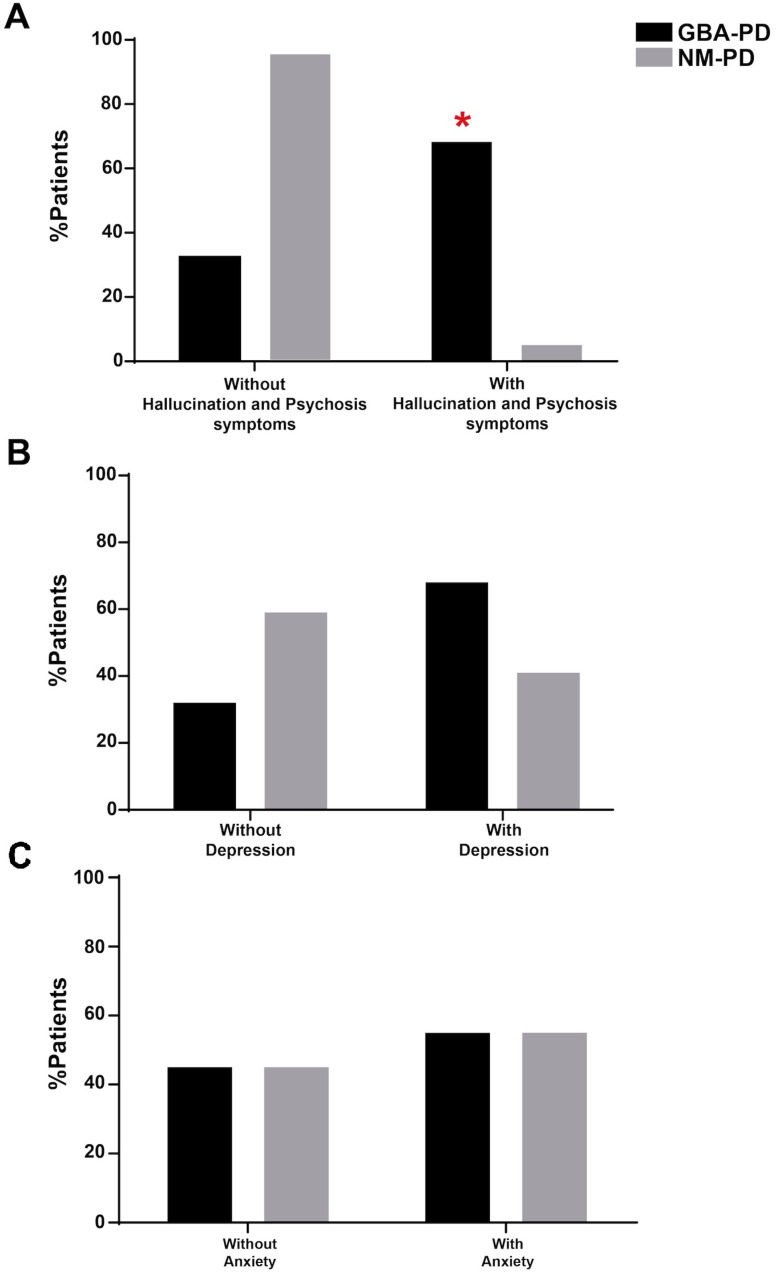
Comparison of PD clinical features between GBA-PD and NM-PD subgroups (**A**–**C**). The GBA-PD cohort showed a significant increment in hallucinations and psychosis symptoms. No differences emerged in depression and anxiety disorders. All data are represented as a percentage and were compared by using the Fisher’s exact test. * *p* < 0.05.

In terms of clinical features, the GBA-PD cohort showed more hallucinations and psychosis symptoms quantified with the MDS-UPDRS item 1.2 (*p* < 0.05, Fisher’s exact test; Figure 2A) and a trend for depression (*p* = 0.069, Figure 2B). No differences emerged in anxiety disorders (Figure 2C).

**Figure 3:**
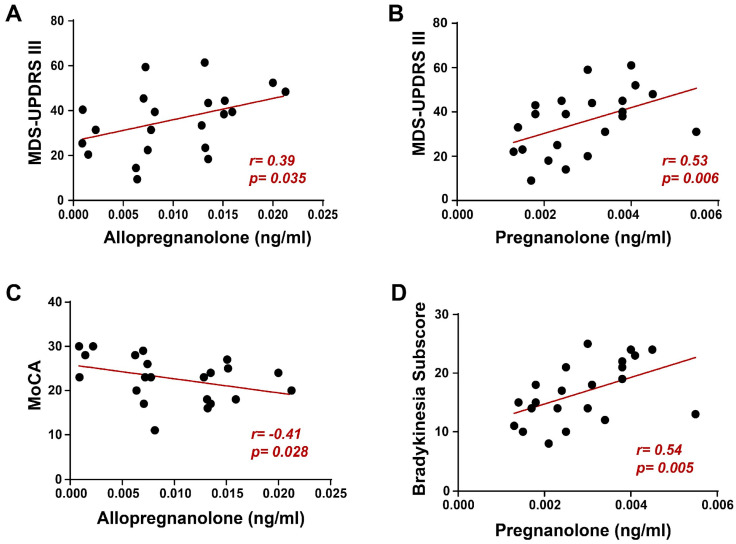
Correlations between neurosteroid serum levels and clinical features in GBA-PD patients. (**A**) In panel A, a significant positive correlation between allopregnanolone serum levels and the MDS-UPDRS III score was found. Also, pregnanolone (**B**) significantly correlates with the MDS-UPDRS III score. In panel (**C**), the negative correlation between allopregnanolone and MoCA proves that GBA-PD patients have a worse cognitive state when higher serum levels of allopregnanolone are present. Conversely, Panel (**D**) showed a significant positive correlation between pregnanolone levels and bradykinesia subscore. The correlations were performed by using the Spearman’s coefficient.

Also, motor aspects of experiences of daily living (MDS-UPDRS part II) were more compromised in GBA-PD patients (*p* < 0.05, Tukey’s *post hoc* test; Table 1), with a trend towards significance for axial manifestations such as balance, walking, freezing, swallowing, and speech (quantified through the postural instability and gait difficulties (PIGD) subscore, *p* = 0.06). Expectedly, both GBA-HC and NM-HC presented significantly lower MDS-UPDRS and, respectively, higher MoCA scores (*p* < 0.05) if compared to GBA-PD and NM-PD. Moreover, we found a positive correlation between allopregnanolone serum levels and the MDS-UPDRS III score in GBA-PD patients (Spearman’s r = 0.39, *p* = 0.035, Figure 3A), suggesting that the differences found in this NS levels between GBA-PD and NM-PD may result in a different clinical phenotype. Surprisingly, we also found a similar result for pregnanolone (Spearman’s r = 0.53, *p* = 0.006, Figure 3B), in which peripheral levels did not differ in the two cohorts of patients. To further investigate the role of these NSs, we tested their correlation with more specific indexes of motor impairment and found that pregnanolone significantly correlated with the bradykinesia subscore (Spearman’s r = 0.54, *p* = 0.005, Figure 3D). Conversely, focusing on non-motor symptoms, we found that allopregnanolone correlated negatively (Spearman’s r = −0.41, *p* = 0.028, Figure 3C) with the MoCA score, suggesting a role of this NS in the cognitive impairment of GBA-PD. Furthermore, we did not find any correlation between allopregnanolone and pregnanolone and clinical features in NM-PD.

## 3. Discussion

GBA gene mutations represent the most frequent genetic determinant of PD, accounting for 5–15% of PD patients worldwide [2,4,6]. Numerous mechanisms have been proposed to explain the GBA-associated neurodegenerative phenotype. However, most related studies have focused on the impact of mutated GCase, especially the loss of enzymatic activity, in neurons, thus neglecting the involvement of other cells composing the CNS [3,21]. In this study, we showed an unprecedented observation of NSs peripheral dysregulation in GBA-PD patients, reporting a significant difference in allopregnanolone levels in the serum of GBA-PD, with higher values in comparison to NM-PD.

The differences in allopregnanolone serum levels between GBA-HC and GBA-PD and between GBA-HC and NM-PD for 5α–DHP could be affected and probably explained by the younger age of subjects composing the GBA-HC cohort [22]. These are because these differences were not confirmed by the comparison with NM-HC. GBA-PD patients have an increased risk of developing more severe motor and non-motor symptoms, including axial impairment, and cognitive and behavioral problems [5]. Considering the link established between behavioral disorders and NSs, we included in our analysis also the evaluation of psychiatric disorders, highlighting their preponderance in GBA-PD. In our study, the GBA-PD cohort showed more hallucinations and psychotic symptoms, a higher impairment in the motor aspects of experiences of daily living, and a trend towards significance for more severe axial PD manifestations compared to NM-PD. These findings were associated with remarkable differences in allopregnanolone serum levels. Thus, it is tentatively suggested that allopregnanolone could be not simply associated but might be possibly involved in the GBA-PD phenotype, even if further studies will be crucial to confirm this preliminary hypothesis.

Our hypothesis can be supported by preclinical evidence showing the involvement of allopregnanolone in the development of psychosis triggered by psychosocial stress [23]. In particular, by reducing the synthesis of allopregnanolone with the 5α-reductase irreversible inhibitor finasteride, the impairment in PPI was alleviated in rats modeling schizophrenia [24,25]. The negative impact of allopregnanolone on PPI was related to the modulation of D1 receptor activity by dopamine [18]. This may explain at least in part the increased risk of developing psychosis and impulse control disorders in GBA patients under dopaminergic chronic oral treatment (particularly with dopamine agonists). Based on these premises, we may hypothesize that the significant increase in the content of allopregnanolone may possibly influence the GBA-PD neuropsychiatric profile. Of course, this hypothesis requires confirmation and that could be further challenged by further studies considering GBA-PD patients possibly treated with 5α-reductase inhibitors.

In addition, we also found an inverse correlation between allopregnanolone serum levels and cognitive status in GBA-PD. In particular, GBA-PD patients with higher levels of allopregnanolone showed lower MoCA scores. This is not surprisingly if we keep in mind the negative effects of chronic benzodiazepines use on cognition [26,27,28,29] and that both benzodiazepines and allopregnanolone modulate positively the γ-aminobutyric acid type A (GABA_A_) receptor complex at the postsynaptic membrane of nerve cells [10]. Indeed, allopregnanolone possesses benzodiazepine-like properties since it’s able to positively modulate GABA_A_ receptors. Allopregnanolone administration was associated with the appearance of memory and learning impairment in rats, and along with other GABA_A_ positive modulators, could potentially cause a cognitive impairment [30]. Recently, GABA_A_ chronic stimulation by benzodiazepines has been associated with cognitive decline during aging in healthy subjects [31]. Although we cannot firmly propose that allopregnanolone might have contributed to the cognitive decline in GBA-PD, the positive relationship we found between allopregnanolone levels and the worsening in the MoCA scores is another piece of evidence supporting the involvement of GABA_A_ receptors in this phenomenon.

In our cohorts, PD led to a reduction of several NSs, as already reported in previous studies [9,10]. In particular, the reduction of allopregnanolone may be related to the neurodegenerative process involving the nigral area [9]. Indeed, the substantia nigra of the human brain expresses high concentrations of allopregnanolone [10]. In this setting, it has been assumed that the decrease of allopregnanolone may represent a biochemical marker of dopaminergic cell loss [9]. This is particularly relevant if we bear in mind the correlation found between the MDS-UPDRS Part III and both allopregnanolone and pregnenolone levels in GBA-PD patients. However, the significant reduction of allopregnanolone in our cohort was only found by comparing GBA-PD with GBA-HC, but with a significant difference in age, which could alternatively explain the statistical finding, and not by comparing age-and gender- matched NM-HC. This finding highlights the need for future studies with age- and gender- matched cohorts of GBA-PD, GBA-HC, and NM-HC in order to better understand the possible role of GBA heterozygous mutations in allopregnanolone expression. This is particularly relevant if considering that GBA is only a risk factor for developing PD and only 10–30% of GBA mutation carriers develop PD eventually [2,6]. Therefore, it is possible that NSs’ dysregulation could be present in GBA carriers that will never develop PD, hallucinations, or psychosis. It was also unexpected to find evidence of a correlation between pregnanolone levels and motor impairment in GBA-PD, especially because no significant difference was found in pregnanolone level measures in NM-PD and GBA-PD. This suggests that NSs able to similarly modulate the GABA_A_ receptors may display a different specificity in their effects, probably related to other different characteristics that are currently unknown.

In summary, the findings reported here represent the first assessment of NSs in GBA-PD patients. We acknowledge the limitations of the study, in particular the small sample size and the assessment of psychosis through the MDS-UPDRS part I and the significant difference in age between GBA-HC and the other three cohorts. Moreover, the significantly higher values for all NSs in both control groups compared to both PD groups and the weak differences in all but 1 out of 6 examined NSs between NM-PD and GBA-PD raise the question if peripheral NS dysregulation is rather a feature of PD pathophysiology or neurodegeneration in general rather than being associated with GBA-PD. In this setting, future studies with larger and age-matched samples and specific scales for the evaluation of neuropsychiatric symptoms will be needed to better assess these issues. Considering that only a minority of GBA gene mutation carriers develop PD, the identification of cofactors capable of impacting the development and progression of the disease, such as NSs, is of undoubtable relevance. From this perspective, understanding the role of NSs in the GBA-PD phenotype may represent a fundamental step to finding new therapeutic targets for this disabling disease.

## 4. Materials and Methods

### 4.1. Patients

Two consecutive cohorts of GBA-PD and NM-PD patients, one GBA-HC, and one further NM-HC cohort were included. The genetic profile was obtained by testing PD patients for 11 pathogenic or likely pathogenic LRKK2 variants and GBA sequences (Table S1). If negative, a next-generation sequencing panel targeting 68 genes involved in PD was performed [2,4,5,6,7]. Each consecutive GBA-PD patient has been matched with a 1:1 pairing method with a consecutive NM-PD subject. In particular, the variables considered for a 1:1 match were: sex, age (tolerance of ±2 years), age at disease onset (with a tolerance of ±2 years), disease duration, and the Charlson Comorbidity Score (CCI) (tolerance of ±2 points). The consecutive GBA-PD patients were selected from an original pull of 60 GBA-PD patients followed at our center, while, for each NM-PD patient, a 1:1 match was performed searching from a pull of 400 consecutive NM-PD subjects [32,33,34]. In particular, the first anonymized patient in a list in random order with matched sex, age, age at disease onset (with a tolerance interval of 2 years), and CCI has been selected. During the pairing method, no other clinical or instrumental data has been considered except the one already mentioned. GBA-HC and NM-HC have been previously screened for the GBA gene through a next-generation sequencing gene panel. The clinical evaluation of GBA-HC showed no premotor PD symptoms (rapid eye movement [REM], sleep behavior disorder [RBD], hyposmia, severe constipation, or mood disorders). These clinical characteristics have been evaluated by retrospectively reviewing medical records and also by interviewing the patients specifically about these premotor symptoms during the clinical assessment. This study was approved by the Ethical Committee of the Area Vasta Emilia Nord (code 2022/0139218), and written informed consent was obtained from each participant included according to the Declaration of Helsinki [24].

### 4.2. Clinical Assessment

PD patients were evaluated through the following clinical scales: the four parts of the MDS-UPDRS [35] and the Hoehn and Yahr scale [36,37], while the GBA-HC and the NM-HC only with the MDS-UPDRS. The following subscores were also extrapolated from the MDS-UPDRS: tremor (items 2.10, 3.15, 3.16, 3.17, 3.18); bradykinesia (items 3.4, 3.5, 3.6, 3.7, 3.8, 3.14); rigidity (items 3.3); PIGD (items 2.12, 2.13, 3.10, 3.11, 3.12); gait (items 3.10, 3.11); dyskinesia (items 4.1, 4.2), fluctuations (items 4.3, 4.4, 4.5, 4.6), hallucinations and psychosis (item 1.2), depressed mood (item 1.3) and anxious mood (item 1.4). In addition, cognitive function was tested in all subjects through the MoCA [38]. The total amount of the dopaminergic treatment was converted into the L-dopa equivalent daily dose (LEDD) using the updated conversion formulae [39]. In addition, the following variables were also collected for NM-PD and GBA-PD cohorts: age at evaluation, age at disease onset, disease duration, and the motor phenotype (akineto-rigid, tremor-dominant).

### 4.3. Quantitative Analysis of NSs in Serum by Liquid Chromatography-Electrospray Tandem Mass Spectrometry (LC-MS/MS)

#### 4.3.1. Chemicals and Reagents

All standards were purchased from Sigma (St. Louis, MO, USA). Internal standards with isotope labeling were the following: 5a-pregnan-3a-ol-20-one-17a,21,21,21-d4 (ALLO-D4) and sodium pregnenolone-17a,21,21,21-d4 sulfate (PS-D4), purchased from Sigma Aldrich. All solvents for high-performance liquid chromatography/electrospray ionization tandem mass spectrometry (HPLC/ESI-MS/MS) were liquid chromatography-mass spectrometry (LC-MS) purity grade, whereas other solvents used for sample preparation were analytical grade (Sigma-Fluka, St. Louis, MO, USA).

#### 4.3.2. Standard Solutions

A stock solution including pregnenolone, pregnenolone sulfate, progesterone, 5α–DHP, allopregnanolone, and pregnanolone was serially diluted with methanol to obtain calibration solutions at ten concentrations. The internal standard solution (IS) was prepared separately. Sample preparations were performed as previously described [40,41,42].

#### 4.3.3. Sample Preparation

Briefly, serum samples obtained from the recruited patients were spiked with IS solution, vortexed, and added with acetonitrile/methanol (70/30; +1.0% formic acid). The samples were then sonicated, centrifuged, and purified on Phree-SPE cartridges to remove endogenous phospholipids. Eluates were concentrated, derivatized with Amplifex Keto Reagent, and transferred in autosampler vials for LC-MS/MS analysis.

#### 4.3.4. LC-MS/MS Analysis

The LC-MS/MS analysis was performed at Centro Interdipartimentale Grandi Strumenti (CIGS), University of Modena and Reggio Emilia. The chromatographic separation was performed on an Agilent 1200 Series Binary Pump (Agilent, Waldbronn, Germany). Mass spectrometric detection was performed using an Agilent QQQ-MS/MS (6410B) triple quadrupole mass analyzer equipped with an ESI ion source (Agilent), operating in the positive mode, as described previously [40].

### 4.4. Statistical Analyses

Continuous variables were expressed as mean (±SD) and median (range), while frequencies and percentages were calculated for categorical variables. First, we tried to compare NS serum levels using a two-way analysis of variance (ANOVA), considering as main factors GBA mutation and PD, but since the Shapiro-Wilk normality test failed and variances in groups were unequal, we secondly used the Kruskal-Wallis test, followed by the Dunn’s test for multiple comparison. Outliers were identified using the Grubbs test and removed before statistical analysis. Clinical continuous variables were compared by using one-way ANOVA followed by Tukey’s post hoc test. Differences in categorical variables between the two groups (hallucinations and psychosis symptoms; depression; anxiety) were assessed by applying Fisher’s exact test. The correlation analyses were performed by calculating the respective Spearman’s r values and their significance level. Data are presented as mean ± standard error of the mean (SEM) or as percentages, and they were regarded as significantly different at *p* < 0.05. Statistical analyses were performed with Sigmaplot 11; (Systat Software, San Jose, CA, USA) and IBM SPSS Statistics for Windows version 20.0 (IBM, Armonk, NY, USA).

## 5. Conclusions

This pilot study provides the first observation of changes in NSs’ peripheral levels in GBA-PD. The involvement of the observed changes in the development of neuro-psychological and motor symptoms of GBA-PD deserves further attention.

## Figures and Tables

**Table 1:** CLINICAL VARIABLES OF THE GBA-PD, NM-PD, GBA-HC, and NM-HC COHORTS.

Variable	GBA-PD (n = 22)	NM-PD (n = 22)	GBA-HC (n = 14)	NM-HC (n = 15)
	No. (%); Mean [SD]; Median {Range}			
Age	63.68 [8.43]; 63.50 {46.00–79.00}	63.05 [8.59]; 62.50 {46.00–79.00}	49.36 [12.58]; 47.50 {30.00–73.00} *°^§^	60.60 [11.77]; 62.00 {41.00–78.00}
Sex, male	11/22 (50%)	11/22 (50%)	8/14 (57.14%)	4/15 (26.66%)
LEDD	704.32 [546.05]; 740.00 {0.00–2297.00}	693.68 [318.02]; 655.00 {157.00–1250.00}	NA	NA
H&Y	2.34 [0.49]; 2.50 {1.00–3.00}	2.45 [0.50]; 2.50 {2.00–4.00}	NA	NA
Disease duration	7.77 [4.38]; 6.50 {2.00–17.00}	7.41 [3.31]; 7.50 {3.00–15.00}	NA	NA
MDS-UPDRS part-I score	13.36 [7.42]; 13.00 {3.00–26.00}	10.23 [6.04]; 9.50 {0.00–22.00}	2.21 [3.01]; 1.00 {0.00–9.00} *°	3.60 [2.29]; 4.00 {0.00–8.00} *°
MDS-UPDRS item 1.2 (hallucinations and psychosis) Present Not present	15/22 (68.18%) 7/22 (31.81%) ^ç^	1/22 (4.54%) 21/22 (95.45%)	0/14 (0.00%) 14/14 (100.00%)	0/15 (0.00%) 15/15 (100.00%)
MDS-UPDRS item 1.3 (depressed mood) Present Not present	15/22 (68.18%) 7/22 (31.81%)	9/22 (40.90%) 13/22 (59.09%)	3/14 (21.42%) 11/14 (78.57%)	0/15 (0.00%) 15/15 (100.00%)
MDS-UPDRS item 1.4 (anxious mood) Present Not present	12/22 (54.54%) 10/22 (45.45%)	12/22 (54.54%) 10/22 (45.45%)	3/14 (21.42%) 11/14 (78.57%)	0/15 (0.00%) 15/15 (100.00%)
MDS-UPDRS part-II score	17.00 [10.52]; 15.00 {2.00–39.00}	10.50 [5.68]; 11.50 {0.00–21.00} ^^^	0.29 [0.469]; 0.00 {0.00–1.00} *°	0.80 [1.424]; 0.00 {0.00–5.00} *°
MDS-UPDRS part-III score	36.00 [13.22]; 38.50 {14.00–61.00}	29.68 [11.77]; 28.00 {13.00–59.00}	0.00 [0.00]; 0.00 {0.00–0.00} *°	0.33 [0.724]; 0.00 {0.00–2.00} *°
MDS-UPDRS part-IV score	4.95 [4.89]; 3.00 {0.00–14.00}	3.36 [3.69]; 3.00 {0.00–12.00}	0.00 [0.00]; 0.00 {0.00–0.00} *°	0.00 [0.00]; 0.00 {0.00–0.00} *°
Tremor Subscore	4.05 [3.34]; 3.50 {0.00–11.00}	3.27 [3.74]; 2.00 {0.00–16.00}	0.00 [0.00]; 0.00 {0.00–0.00} *°	0.27 [0.704]; 0.00 {0.00–2.00} *°
Bradykinesia subscore	17.86 [5.52]; 17.00 {8.00–26.00}	14.95 [5.54]; 14.00 {6.00–22.00}	0.00 [0.00]; 0.00 {0.00–0.00} *°	0.00 [0.00]; 0.00 {0.00–0.00} *°
Rigidity subscore	5.27 [2.89]; 5.00 {1.00–12.00}	4.64 [2.88]; 4.00 {0.00–11.00}	0.00 [0.00]; 0.00 {0.00–0.00} *°	0.00 [0.00]; 0.00 {0.00–0.00} *°
PIGD subscore	7.14 [4.70]; 7.00 {0.00–19.00}	4.68 [3.34]; 4.00 {0.00–15.00}	0.00 [0.00]; 0.00 {0.00–0.00} *°	0.00 [0.00]; 0.00 {0.00–0.00} *°
Gait subscore	2.59 [1.81]; 2.00 {0.00–7.00}	1.73 [1.42]; 1.00 {0.00–6.00}	0.00 [0.00]; 0.00 {0.00–0.00} *°	0.07 [0.258]; 0.00 {0.00–0.00} *°
Fluctuations subscore	3.55 [3.40]; 3.00 {0.00–10.00}	2.68 [2.80]; 2.50 {0.00–8.00}	0.00 [0.00]; 0.00 {0.00–0.00} *°	0.00 [0.00]; 0.00 {0.00–0.00} *°
Dyskinesia subscore	0.62 [1.79]; 0.00 {0.00–4.00}	0.68 [1.32]; 0.00 {0.00–4.00}	0.00 [0.00]; 0.00 {0.00–0.00} *°	0.00 [0.00]; 0.00 {0.00–0.00} *°
MoCA	22.73 [5.10]; 23.00 {11.00–30.00}	21.82 [5.43]; 24.00 {12.00–28.00}	29.79 [0.80]; 30.00 {27.00–30.00} *°	29.40 [0.98]; 30.00 {27.00–30.00} *°
Motor phenotype Akineto-rigid Tremor	13/22 (59.09%) 9/22 (40.90%)	9/22 (40.90%) 13/22 (59.09%)	NA	NA

Abbreviations: H&Y: Hoehn and Yahr stage; GBA-HC: glucocerebrosidase mutated healthy controls; GBA-PD: glucocerebrosidase mutated with Parkinson’s Disease; LEDD: L-dopa equivalent daily dose; MoCA: Montreal Cognitive Assessment; MDS-UPDRS: Movement Disorder Society revision of the Unified Parkinson’s Disease Rating Scale; NM-HC: non-mutated healthy controls; NM-PD: non-mutated with Parkinson’s Disease; PIGD: postural instability and gait difficulties. * *p* < 0.005 compared with GBA-PD subgroup, ° *p* < 0.005 compared with NM-PD subgroup, ^§^ *p* < 0.05 compared with NM-HC subgroup, ^ *p* < 0.05 compared with GBA-PD subgroup (one-way analysis of variance followed by Tukey’s *post hoc* test); ^ç^ *p* < 0.05 compared with NM-PD subgroup (Fisher’s exact test).

## Data Availability

The data presented in this study are available on request from F.V. and require a new application and approval by the Ethical Committee of the Area Vasta Emilia Nord, in agreement with the Italian rules.

## Supplementary Materials

The following supporting information can be downloaded at: https://www.mdpi.com/article/10.3390/biom14081022/s1. Table S1. List of GBA variants identified in GBA-PD and GBA-C groups.
