# Supplementary material for: Neurosteroid Levels in GBA Mutated and Non-Mutated Parkinson’s Disease: A Possible Factor Influencing Clinical Phenotype?
Source: Biomolecules. 2024 Aug 17;14(8):1022. doi: 10.3390/biom14081022 (PMC11352262; doi:10.3390/biom14081022)
Supplement: Supplementary file 1 [file biomolecules-14-01022-s001.zip › biomolecules-3145308-supplementary.pdf]

SUPPLEMENTARY TABLE 1. List of GBA variants identified in GBA-PD and GBA-C groups			
VARIANT	**GBA-PD (n= 22)**<br>**No. (%)**	**GBA-C (n= 14)**<br>**No. (%)**	**SEVERITY OF MUTATION**
p.(Thr408Met)	6 (27.27%)	2 (14.28%)	Risk factor
p.(Asn409Ser)	3 (13.63%)	4 (28.57%)	Mild
p.(Glu365Lys) + p.(Thr408Met)	1 (4.54%)	-	Mild
p.(Leu483Pro) + p.(Glu365Lys)	1 (4.54%)	-	Severe
p.(His294Gln) + p.(Asp448His)	1 (4.54%)	-	Severe
p.(Arg86*)	1 (4.54%)	3 (21.42%)	Severe
p.(Glu365Lys)	3 (13.63%)	-	Risk factor
(p. Asn227Ser)	3 (13.63%)	1 (7.14%)	Severe
p.(Leu483Pro)	2 (9.09%)	2 (14.28%)	Severe
p.(Asp438Asn)	1 (4.54%)	-	Severe
c.115+1G>A	-	2 (14.29%)	Severe
